# Reduced sensitivity to tactile stimuli associated with physical and mental disorders: A monozygotic twin study

**DOI:** 10.1038/s41598-026-56098-0

**Published:** 2026-06-10

**Authors:** Saito Sakaguchi, Yusuke Morito, Masashi Konyo, Dousatsu Sakata, Kanako Akada, Mikio Watanabe, Norio Sakai, Yusuke Hara, Osaka Twin Research Group, Dousatsu Sakata, Norio Sakai, Masanori Takahashi, Teiji Nishio, Kei Kamide, Shinji Kihara, Hiroko Watanabe

**Affiliations:** 1 MIRAI Technology Institute, Shiseido Co., Ltd., Yokohama, Japan; 2 Grad. Sch. of Information Sciences, Tohoku University, Sendai, Japan; 3 Center for Twin Research, Graduate School of Medicine, The University of Osaka, Osaka, Japan; 4 Department of Clinical Laboratory and Biomedical Sciences, Graduate School of Medicine, The University of Osaka, Osaka, Japan

**Keywords:** Tactile perception, Tactile sensitivity, Environmental factors, Monozygotic design, Quality of life, Sensory information, Genetics, Psychology, Health care, Signs and symptoms

## Abstract

Tactile sensitivity is crucial for perceiving subtle external stimuli and maintaining a high quality of life. While individual differences in tactile sensitivity are influenced by local factors, such as skin condition and nerve density, the relationships between tactile sensitivity and systemic factors, such as physical and mental health, remain unclear. This study investigated how systemic conditions are related to tactile sensitivity, excluding genetic influences, by analyzing 25 pairs of monozygotic (MZ) twins. Tactile thresholds were measured using suction stimuli on the cheek and compared with psychophysiological data from questionnaires and objective measurements. The results revealed that 18 twin pairs presented similar thresholds, indicating a genetic influence. However, in 7 twin pairs with differing thresholds, reduced tactile sensitivity was associated with greater physical and mental disorders, such as fatigue and anxiety. These findings suggest that tactile sensitivity is influenced not only by genetic factors but also by systemic health conditions that are shaped by lifestyle. Furthermore, impaired tactile sensitivity may reflect broader physical and mental health issues, making it a potential marker for preventive health interventions. This study provides insights into the complex interplay between tactile perception and systemic health, paving the way for tactile interventions aimed at enhancing the quality of life.

**Supplementary Information:**

The online version contains supplementary material available at 10.1038/s41598-026-56098-0.

## Introduction

The ability to perceive information from outside the body is essential for maintaining a high quality of life (QOL). There is an increasing shift in healthcare from treating diseases to focusing on prevention and the extension of healthy life expectancy while maintaining a high QOL^1–3^. A decline in sensory function can lead to issues such as loneliness, isolation, dependence, and communication disorders^4–7^. For example, the loss of tactile sensation, in combination with vision impairment, significantly decreases the QOL^8^. The range of tactile functions is broad and closely linked to our daily lives, providing essential information for precise hand movements, managing psychological distress and pain, and facilitating emotional communication^9–11^.

Tactile perception, quantified by thresholds for light touch (monofilament), vibrotactile stimuli, and spatial acuity, is known to decline with age and shows significant variability even among younger individuals^12–14^. Individual differences in quantified sensitivity likely affect the quality of information received from the environment. By understanding the origins of these differences in skin sensation (e.g., innate factors or environmental influences such as lifestyle) and its effects (e.g., physical and mental states or cognitive behaviors), effective interventions can be developed, highlighting the importance of tactile perception in maintaining the QOL.

The relationship between localized physical conditions and tactile perception has gradually been elucidated through experimental studies. Neurophysiological observations indicate correlations between bump detection^15^ and roughness perception^16^ with peripheral mechanoreceptor density, and similar inferences have been made regarding nerve density^17^. Additionally, skin conformance has been shown to partially explain spatial acuity^18^ and pressure detection thresholds^19^. Moisturizing interventions targeting the mechanical properties of the skin have been shown to restore tactile perceptual abilities^20^^,^^16^^,^^21^^,^^22^.

The relationships between individual differences in tactile perception and systemic factors (such as lifestyle, attitudes, and body and mental states) beyond peripheral skin conditions remain unclear. While exploratory studies have examined the connections between tactile perception and systemic traits, such as empathic concern^23^, or between tactile hypoesthesia and major depressive symptoms^24^, a comprehensive understanding of these connections remains lacking. One reason for this shortcoming is that conventional studies comparing unrelated individuals have difficulty separating environmentally driven variation in tactile perception from genetically mediated individual differences. Consequently, observed associations with systemic factors may reflect underlying biological predispositions rather than experiential influences. By minimizing the influence of genetic and constitutional variability, it becomes possible to clarify the relationships between tactile sensitivity and potentially modifiable physiological or psychological states within the complex network of systemic factors. Identifying such modifiable associations is essential for guiding the development of effective interventions aimed at maintaining or improving the QOL through tactile function.

To address these challenges, we employed a monozygotic (MZ) twin design to explore the physical and mental conditions associated with tactile sensitivity while eliminating genetic factors. Biologically, MZ twins share identical DNA sequences, allowing us to target experiential differences while maintaining many nongenetic factors (e.g., age, upbringing, and socioeconomic influences) relatively constant. This design enables us to determine whether measured experiences or exposures are truly driven by the environment or experience without being confined by genetic factors. Given the substantial individual variability in tactile sensitivity, this approach is particularly useful.

In this study, we aim to investigate the physical and mental conditions associated with tactile sensitivity in an MZ twin design by parsing environmentally driven (nongenetic) contributions. This study provides a novel approach to understanding the complex interactions between tactile perception and overall health, potentially leading to more effective interventions for improving the QOL through enhanced tactile sensitivity.

We measured tactile sensitivity in a sample of MZ twins and examined the differences in sensitivity across twin pairs. The cheek was chosen as the testing site because of its high tactile sensitivity among hairy areas^25^ and its potential involvement in perceptual and expressive functions related to the QOL^26^^,^^27^. We used suction stimulation to quantify the tactile sensitivity through the skin, as this method facilitates the observation of the mechanical properties of the superficial skin layer as opposed to the deeper layers of muscle and fat^28,29^. We then compared the differences in tactile sensitivity within MZ pairs with data obtained from objective physical measurements and questionnaire responses assessing overall physical and mental health. This approach enabled us to identify characteristics representing physical and mental conditions associated with differences in tactile sensitivity within MZ pairs.

## Results

### Age and tactile sensitivity

**Fig. 1:**
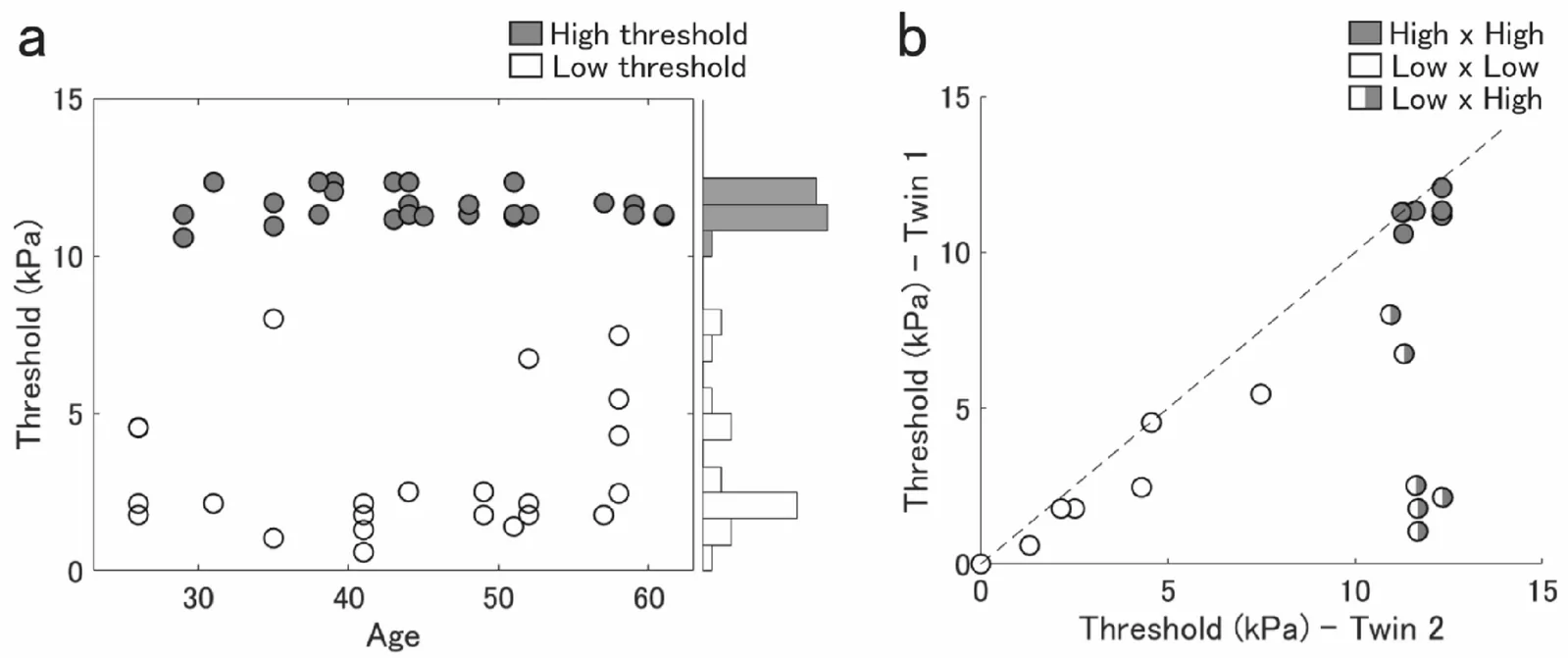
Despite individual variability, monozygotic twin pairs presented similar tactile thresholds. (**a**) Relationship between age and tactile threshold (kPa) for 50 participants. Lower thresholds indicate greater sensitivity to tactile stimuli. On the basis of a histogram color-coded by the median, high thresholds are shown in gray, and low thresholds are shown in white. Substantial individual differences are evident, even among participants of the same age. (**b**) Cross-twin relation: in 10 twin pairs, both twins presented high thresholds; in 8 pairs, both twins presented low thresholds; and in 7 twin pairs, one twin presented a high threshold and the other a low threshold.

Tactile sensitivity was assessed by calculating the tactile threshold in response to negative pressure, whereby a lower threshold corresponds to a greater ability to perceive smaller tactile stimuli. The representative threshold values for all the participants are shown in Fig. 1a. The tactile thresholds were broadly distributed across all trials, with a median value of 10.9 (IQR: 2.5–11.6 kPa). No significant association was observed between age and tactile thresholds in linear mixed-effects models including twin pair as a random intercept (β = 0.039, *p* = 0.621, 95% CI [-0.113, 0.191]). The data distribution appeared to be bimodal, suggesting a division into groups with higher and lower thresholds around the median value, as illustrated through histograms. Both higher and lower threshold values were observed across all age (years) groups (20s, 30s, 40s, and ≥50s; the single pair of twins in their 60s was included in the ≥50 group).

### Twin distribution of tactile sensitivity

The cross-twin relations are shown in Fig. 1b, where the higher tactile threshold is plotted on the horizontal axis. Most pairs were plotted on the line of identity, indicating that twin pairs generally share similar thresholds. Notably, among the 25 twin pairs, 18 had a smaller difference in median values within the twin pair than the average individual threshold variation (difference between maximum and minimum values) of 2.57, suggesting that individuals with the same genetic information tend to have similar thresholds.

When the thresholds were divided into higher and lower groups on the basis of the histograms, 10 of the 18 similar-threshold pairs presented higher thresholds, and 8 pairs presented lower thresholds. In contrast, 7 pairs were divided into groups with higher and lower thresholds. The background characteristics of the pairs with different thresholds are listed in Table 1. The average age of the similar-threshold pairs was 45±11 years, and the average age at which the pair members started living separately was 22±8.3 years, whereas for the different-threshold pairs, the average age was 44±9 years, and the average age of separation was 25±5.4 years. There was no trend that indicated that the difference in thresholds was due to earlier separation. We also confirmed that there were no substantial differences in the history of cosmetic procedures on the face or smoking history among these pairs. UV exposure history was also assessed (Supplementary Fig. S1), which revealed that two pairs had nearly identical UV exposure histories, whereas in four pairs, the twin with the higher tactile threshold had greater UV exposure across most age periods.

**Table 1:** Background factors of the different threshold pairs.

	Twins with different thresholds	
	Higher threshold	Lower threshold
Threshold (kPa)	11.5±0.4	3.4±2.6
Age (years)	43.6±9.3	
Age at separation	24.9±5.4	
Height (cm)	159.3±3.0	158.4±2.7
Weight (kg)	56.5±5.5	54.9±3.6
BMI (kg/m^2^)	22.3±2.1	21.9±1.7
Smoking in the past month	None	None
Cosmetic treatments in the past 3 years	Chemical peeling (n=1)	None

In summary, among the MZ twins, more than half of the pairs presented similar tactile thresholds, thus indicating a substantial contribution of genetic factors to tactile sensitivity. Seven of the 25 pairs presented markedly different tactile perceptual abilities.

### Psychophysical characteristics and tactile sensitivity

To first assess population-level associations, we examined the relationships between tactile thresholds and 56 psychophysical characteristics derived from questionnaires and device measurements across all participants (n = 50) using linear mixed-effects models accounting for twin-pair clustering. None of the tested characteristics showed a statistically significant association with tactile thresholds after false discovery rate correction, and none reached the predefined large effect size threshold (Supplementary Table S1). These findings indicate that tactile thresholds were not strongly associated with the examined characteristics at the whole-sample level.

**Fig. 2:**
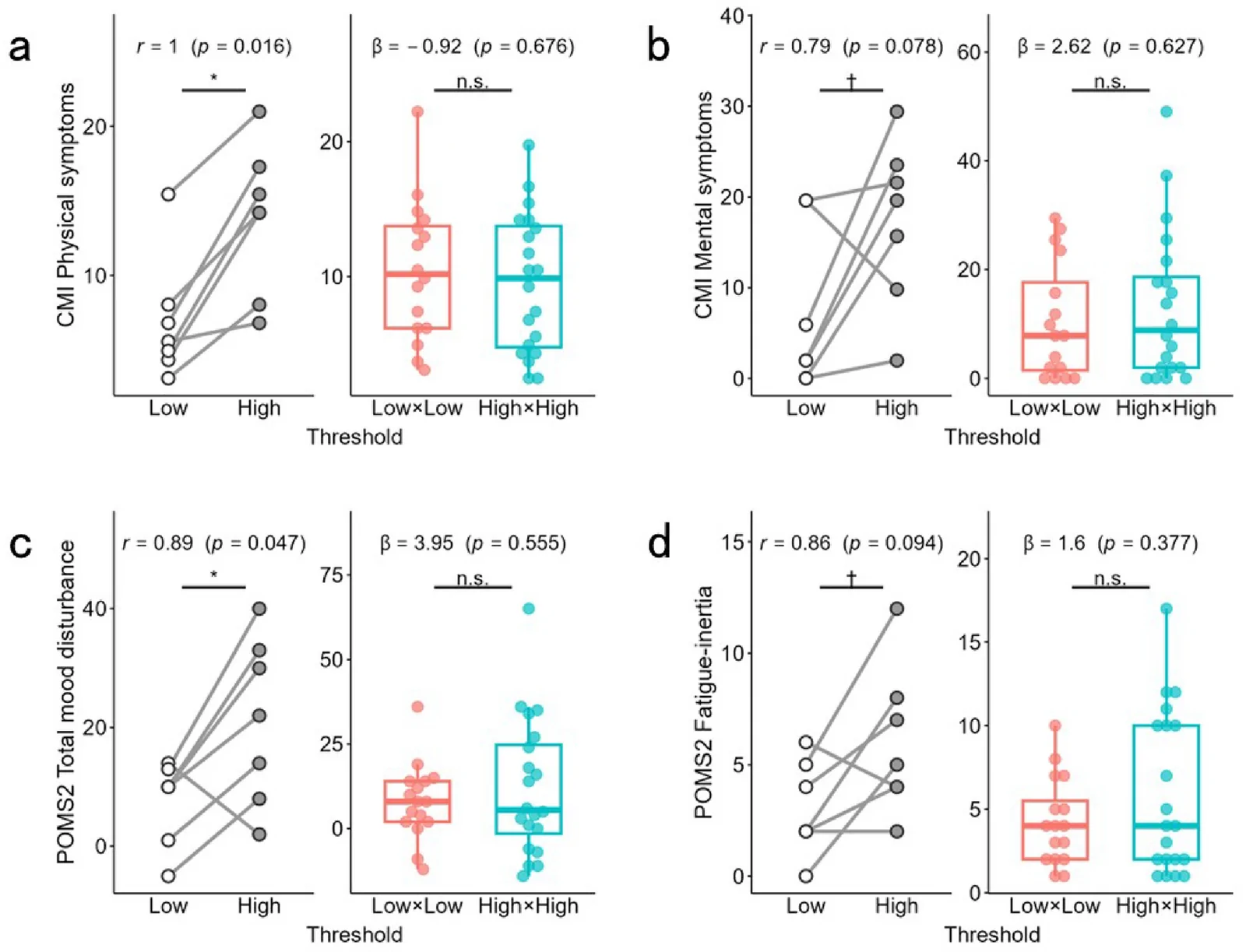
Reduced tactile sensitivity, excluding genetic factors, is associated with the perception of physical and mental disorders and negative moods. This figure illustrates representative psychophysical characteristics related to threshold differences in the 7 different-threshold twin pairs. The paired plots connected by lines in the figure on the left represent twin pairs. Regardless of genetic factors, higher thresholds are associated with (**a**) physical disorders (CMI_Physical symptoms), (**b**) mental disorders (CMI_Mental symptoms), (**c**) negative mood (POMS2_Total mood disturbance), and (**d**) fatigue (POMS2_Fatigue-inertia). The box plots on the right show the results for 18 twin pairs with similar thresholds. There were no significant differences in these scores between the 10 twin pairs with low thresholds (n = 20, low x low) and the 8 twin pairs with high thresholds (n = 16, high x high), indicating that the relationship between tactile thresholds and characteristics such as physical and mental disorders is not genetically determined. *: *p* < 0.05, †: *p* < 0.10, n.s.: not significant.

We therefore focused on MZ twin pairs with different tactile thresholds and explored the associations between tactile perceptual ability and psychophysical characteristics as such within-pair differences provide a particularly informative opportunity to examine these relationships while minimizing the influence of a shared genetic background. Within these pairs, paired comparisons between twins with lower and higher thresholds were conducted using the Wilcoxon signed-rank test. The characteristics associated with tactile thresholds are summarized in Table 2, with representative examples shown in Fig. 2. For the profile of mood states second edition (POMS2) instrument, which provides a comprehensive assessment of the negative mood state, the total mood disturbance score was significantly higher for participants with higher tactile thresholds within twin pairs than for those with lower thresholds (TMD: *r* = 0.893, *p* = 0.047; Fig. 2). The POMS2 subscale results for anger–hostility (AH) and fatigue–inertia (FI) indicated a tendency toward higher tactile thresholds for those with “anger and resentment toward others” and “feelings of fatigue and decreased motivation or vitality”. In addition, the Cornell Medical Index (CMI) physical symptoms, which indicate the degree of physical disorders, including “fatigue level” and “gastrointestinal discomfort,” were considerably greater for participants with higher tactile thresholds within twin pairs (physical symptoms: *r* = 1.00, *p* = 0.016; Fig. 2). The physical subscales related to the digestive tract and genitourinary system suggested a tendency for those with higher tactile thresholds to feel disorders in areas such as “appetite and digestion,” “urination,” and “menstruation”. The CMI mental symptoms, which indicate the degree of psychological disorders, such as “depression,” “anxiety,” and “hypersensitivity,” also tended to be greater in participants with lower tactile sensitivity (mental symptoms: *r* = 0.786, *p* = 0.078; Fig. 2). Furthermore, the Cutometer^®^-measured gloss elasticity (R2) value revealed a tendency for participants with lower skin elasticity within twin pairs to have lower tactile sensitivity.

**Table 2:** Psychophysical characteristics related to tactile threshold differences in 7 monozygotic twin pairs.

		**Lower threshold**		**Higher threshold**				
**Measure**	**Variable**	**Median**	**IQR**	**Median**	**IQR**	**Median** **difference**	***p***	**Effect** **size (*r*)**
CMI	Physical symptoms	5.56	2.78	14.20	5.25	6.17	0.016	1.000
	Digestive tract	3.57	1.79	10.71	7.14	7.14	0.062	0.905
	Mental symptoms	1.96	11.77	19.61	9.80	15.69	0.078	0.786
	Genitourinary system	15.39	15.39	23.08	7.69	15.39	0.094	0.750
POMS2	Total mood disturbance (TMD)	10	6	22	20.5	13	0.047	0.893
	Anger-hostility (AH)	2	2.5	5	3.5	3	0.078	0.786
	Fatigue-inertia (FI)	3	1	5	6	2	0.094	0.857
Cutometer	Gross elasticity (R2)	0.680	0.097	0.637	0.077	-0.023	0.094	-0.714

Results of paired comparisons within twin pairs using the Wilcoxon signed-rank test, including median (IQR), median paired differences (high − low), rank-biserial correlation effect sizes (*r*), and exact p values (*p*).

To further evaluate whether these associations might reflect genetic influences, we examined twin pairs with similar tactile thresholds using linear mixed-effects models. The psychophysical characteristics that showed associations with threshold differences within pairs (Table 2) were compared between twin pairs with uniformly high thresholds and those with uniformly low thresholds. Both groups exhibited considerable variability in these characteristics, and the effect of group (high vs. low tactile threshold) was not statistically significant for any of the examined variables (all *p* > 0.05; Fig. 2**;** see Supplementary Table S2 for detailed group-level descriptive statistics).

In short, an association was found between changes in tactile thresholds that occur within pairs of MZ twins, i.e., due to environmental factors, and the physical and mental disorders experienced by participants.

## Discussion

We explored the distribution of tactile sensitivity in MZ twin pairs and the relationships between tactile sensitivity, excluding genetic factors, and psychophysical characteristics. Despite considerable individual variability in tactile sensitivity in response to skin stimuli, the majority of the MZ twin pairs presented similar sensitivities, indicating that a large portion of tactile sensitivity is influenced by genetic factors. Expanding on this finding, we investigated the different threshold pairs and demonstrated a connection between variations in tactile sensitivity due to environmental factors and the self-perception of physical and mental disorders. This study underscores the importance of tactile perception in maintaining the quality of life by providing new evidence on how skin sensation is related to mental, physical, and cognitive behaviors, suggesting avenues for more effective holistic interventions.

Tactile sensitivity to deformation of the superficial skin layer, as measured by suction stimulation, is thought to be influenced by substantial genetic factors. Consistent with previous studies that demonstrated variability in sensitivity to superficial skin stimulation without a linear age effect^19^, we found that thresholds varied independently of age, with individual-specific values. Remarkably, most twin pairs (18 of 25 pairs) presented less intrapair variability in thresholds between twins than the variability observed within individuals (i.e., similar thresholds within pairs), indicating the substantial impact of genetic factors. Previous studies focusing on the heritability of mechanical skin stimulation in MZ and dizygotic twins reported a heritability of 52% for vibration detection thresholds^30^ and 55% for pain perception following the perpendicular application of mechanical force to the skin^31^, indicating the extent of genetic influence. Our findings align with these results, suggesting that genetic factors substantially affect tactile sensitivity to mechanical stimuli, regardless of the type of skin deformation.

When genetic factors were excluded, the relationship between self-perception of physical and mental disorders and tactile sensitivity became clear despite the complex interplay of holistic systems. Despite the considerable genetic factors observed, we also observed a substantial difference in tactile thresholds within twin pairs in a sample of 7 pairs (14 individuals). We were able to implement an intrapair design that clearly excluded genetic factors. In similar prior studies targeting participants without physical disabilities or skin disease damage, a small number of individuals exhibited higher thresholds (i.e., failed to perceive stimuli below 10 kPa)^19^^,^^32^. When the participants in our study were grouped on the basis of a bimodal distribution of thresholds, those with higher thresholds could be categorized as having “clearly poor tactile sensitivity.” Our findings suggest an association between greater awareness of physical and mental symptoms, including fatigue, and reduced tactile sensitivity. This relationship appears to be formed by clear environmental factors, such as lifestyle and daily stress, rather than genetic factors. Since the difference in thresholds that occurred in twin pairs did not depend on the age of separation, such phenomena may have occurred because of the effects of environmental factors, even at a young age.

While fatigue-related sensory impairments have been demonstrated experimentally, our findings suggest that self-perceived psychophysical disorders may also influence tactile perception. Previous studies have shown that fatigue induced by mental or physical tasks can impair cognitive and motor performance that relies on sensory information^33–36^. In addition, muscle fatigue has been reported to increase the tactile threshold for light touch^37^ and reduce proprioceptive acuity^38^, even if fatigue is induced away from the tactile stimulus site. These findings suggest that fatigue-related states may influence sensory processing through central mechanisms affecting afferent feedback. Consistent with this framework, our results indicate that fatigue-related states, including self-perceived physical and mental symptoms, are associated with reduced tactile sensitivity, extending previous findings that have reported mainly fatigue-related effects on performance and proprioceptive function pertaining to the detectability of subtle mechanical stimulation of the superficial skin layer^28,29^, which is likely mediated by cutaneous mechanoreception. Furthermore, whereas earlier research primarily examined acute responses following experimentally induced short-term fatigue, our results further suggest that subjective states in everyday life, or potentially more persistent psychophysical symptoms and fatigue, may also be associated with reduced tactile sensitivity. Mechanisms underlying experimentally induced mental fatigue have often been discussed in terms of decreased engagement, depletion of brain energy resources, and reduced cognitive control due to attentional dispersion^39,33,34,40^. The present findings raise the possibility that such mechanisms, typically considered in the context of short-term fatigue, may also be relevant from the perspective of more sustained states of central nervous system processing, which could contribute to individual differences in tactile sensitivity. Such environmentally associated differences may therefore not be limited to the face and could also occur at other body sites.

Diminished tactile sensitivity may serve as a sensory‒perceptual indicator related to everyday mental health. Recent evidence in psychopathology has highlighted the critical role of sensory functions in terms of mental health^41^, with many studies primarily focusing on visual and auditory modalities and reporting that sensory deficits are associated with anxiety, depressive symptoms, and feelings of hopelessness^42–44^. Given the role of cognitive processes in the brain, sensory impairments have been suggested as independent factors contributing to cognitive decline and psychopathologies such as depression, anxiety, and fatigue^45–49^. Considering that items related to tactile differences were associated with self-perception of physical and mental disorders, including feeling weary, exhausted, tired, angry, or anxious, these findings complement those of previous studies by extending the link between sensory functions and mental health to the tactile modality, suggesting that tactile sensitivity may serve as a precursor to negative mental health conditions. Broadly, tactile sensory dysfunction has also been demonstrated in major depressive disorder, which is characterized by mood dysregulation, fatigue, and anhedonia. Specific examples of tactile sensory dysfunction include deficits in tactile learning tasks^50^, reduced pain-free electroperception and increased sensitivity to pain perception^51^, reduced temperature sensitivity^51,52^, hypoesthesia^24^, and abnormal interoceptive sensations^53^. Although the tactile sensitivity measured in the present study involved subtle skin sensations that were neither painful nor itchy, such changes in sensory processing may represent milder forms of tactile dysfunction that, if sustained, could be related to mechanisms underlying these previously reported abnormalities. These findings raise the possibility that monitoring changes in tactile sensitivity may aid in the early detection and treatment of mental health issues.

The relationship between the mechanical properties of the skin at the site of tactile stimulation and tactile sensitivity was consistent with findings from previous reports. Although the statistical analysis suggested that the effect size was smaller than that of the other significant characteristics, there was a trend indicating that skin with more elasticity had better sensitivity. Previous studies that focused on the role of the skin using suction methods have shown that skin that is more easily deformed in response to periodic stimuli (i.e., skin with more elasticity) is more perceptible^19^ and that differences in tactile sensitivity, even among individuals of the same age, are related to skin elasticity^32^. Our findings emphasize the impact of differences in skin mechanical properties, induced by environmental factors, on tactile acuity.

The limitations of this study include the small sample size and the absence of a longitudinal approach. The majority of tactile thresholds within twin pairs were similar, and the experimental design using MZ twins to eliminate genetic factors was applicable to only 7 pairs (14 participants). While this was an interesting result suggesting the magnitude of the effect of genetic factors, recruiting more MZ twin pairs could facilitate a more detailed visualization of within-pair similarity distributions, and the data obtained from within-twin comparisons would likely be more valuable. The scarcity of discordant threshold pairs in this study could be due to the substantial effect of genetic factors on tactile perception or because the degree of perceived physical and mental disorders was similar within the twin pairs. For clarity, a longitudinal approach—essentially, rerecruiting the same twin pairs for a follow-up study—would be necessary to capture changes in disorder perception scores and thresholds, potentially revealing a clearer relationship. Notably, self-perception of physical and mental states such as discomfort and fatigue was obtained through questionnaires. Therefore, it remains unclear whether these states are short- or long-term and whether there are objective bodily changes underlying perceived disorders. Another limitation of this study is that only female participants were included; therefore, the findings may not be generalizable to males, and potential sex differences in tactile sensitivity could not be assessed.

Finally, these findings suggest avenues for future studies. The extracted threshold discrepancy pairs suggest the existence of individuals who are genetically predisposed to fatigue and sensory perception. Identifying the genetic factors that characterize these individuals may contribute to clinical applications and improvements in the quality of life through a genetic approach. Furthermore, the potential impact of interoceptive disturbances on exteroceptive perception is of interest^41^, as our study revealed that fatigue and physical disorders, particularly in the urinary and digestive systems (interoceptive elements), were related to reduced skin sensitivity. Previous research has shown that changes in interoceptive signals (e.g., heart rate and cardiac cycle timing) can alter exteroceptive sensitivity. These observations raise the possibility that bodily disorders that alter interoceptive signals may also influence exteroceptive perception through this pathway. Moreover, the impact of sensory information on mental health and psychiatric disorders remains underexplored. Sensory impairments may independently contribute to depression and cognitive decline, leading to a loss of interest in daily activities and an inability to experience pleasure^46^^,^^44^. Interventions using sensory aids for visual and auditory senses may be feasible^45^; similarly, restoring tactile sensitivity through skin interventions could improve mental health^21^^,^^16^. Additionally, exploring affective tactile stimuli could be insightful. The perception of pleasant touch varies with self-reported mental health^54^, and it is debated whether difficulties in affective touch perception are a consequence of or contributing factor to mental health disorders^55^. Comparing discriminative touch with socially oriented affective touch, accompanied by longitudinal studies on mental health, could enhance our understanding of the interaction between sensory information and central processing in tactile perception and cognition.

## Methods

### Participants

We used monozygotic twin pairs to ensure the genetic uniformity needed for our study. A total of twenty-five Japanese female MZ twin pairs, whose ages ranged from 26 to 61 years (mean = 44.52 years; standard deviation = 10.24 years), were recruited from a registry established by the Center for Twin Research at the University of Osaka^56^. The inclusion criterion was adult female monozygotic twin pairs. Written informed consent was obtained from all the subjects before inclusion in the study. Both the Ethics Committee of the University of Osaka and Shiseido Co., Ltd., approved the study protocol (Nos. 696-8 and 22204-2). All methods were performed in accordance with the Declaration of Helsinki and relevant guidelines and regulations. The zygosity of the twins was confirmed by perfectly matching 15 short tandem repeat loci using a PowerPlex^®^ 16 System (Promega). The exclusion criteria included pregnancy or breastfeeding; the presence of tattoos or dermatological diseases at the measurement site; a history of surgical procedures at the measurement site; and any other condition judged by the investigators to make the participant unsuitable for the study.

### Apparatus

We used a previously developed device to deliver quantitative suction stimulation to the skin^57^^,^^58^ measuring tactile sensitivity while eliminating the influence of deep muscle or fat^28^^,^^29^. A voice‒coil linear actuator (H2W Technologies, VMS30-090-LB-1) compressed and expanded the air in an air cylinder (SMC, MQQLL30-100DM). The oscillation generated by the actuator was transmitted via an air tube to a contactor, which applied suction pressure to the skin. The contactor had a 2 mm diameter suction hole in the center. During the measurements, a spring-loaded contact force adjustment mechanism maintained a constant pressing force on the skin. A pressure sensor (PISCO, VUS11-AR) was attached to the air tube to quantify the pressure applied to the cheek.

### Experimental procedures

The described experiment was conducted as part of a larger skin physiology study. Only the procedures relevant to the present study are described below.

Each participant was tested individually, and all measurements were performed on the same day. All measurements were conducted under identical environmental conditions across participants. The tactile sensitivity measurements were performed by the same experimenter for all participants and were conducted individually in a private testing room. The order of testing within each twin pair was randomized, but the two members of each pair were measured consecutively under identical conditions, with the second twin tested immediately after completion of the first twin’s measurements.

The experimental procedure was as follows. First, basic physiological measurements were obtained. Participants then washed their faces, followed by a 15 min acclimatization period. Skin measurements were subsequently conducted, after which tactile sensitivity was assessed. Finally, the participants completed the questionnaires. Although not analyzed in the present study, additional skin measurements (e.g., skin color and texture) were obtained using probe-based devices. No procedures that could alter the mechanical properties of the skin at the test site (e.g., gel application or stratum corneum stripping) were performed prior to the tactile measurements. Tactile sensitivity was measured 2 h after face washing. The tactile sensitivity assessment required approximately 15 min per participant.

### Measurement of tactile thresholds

**Fig. 3:**
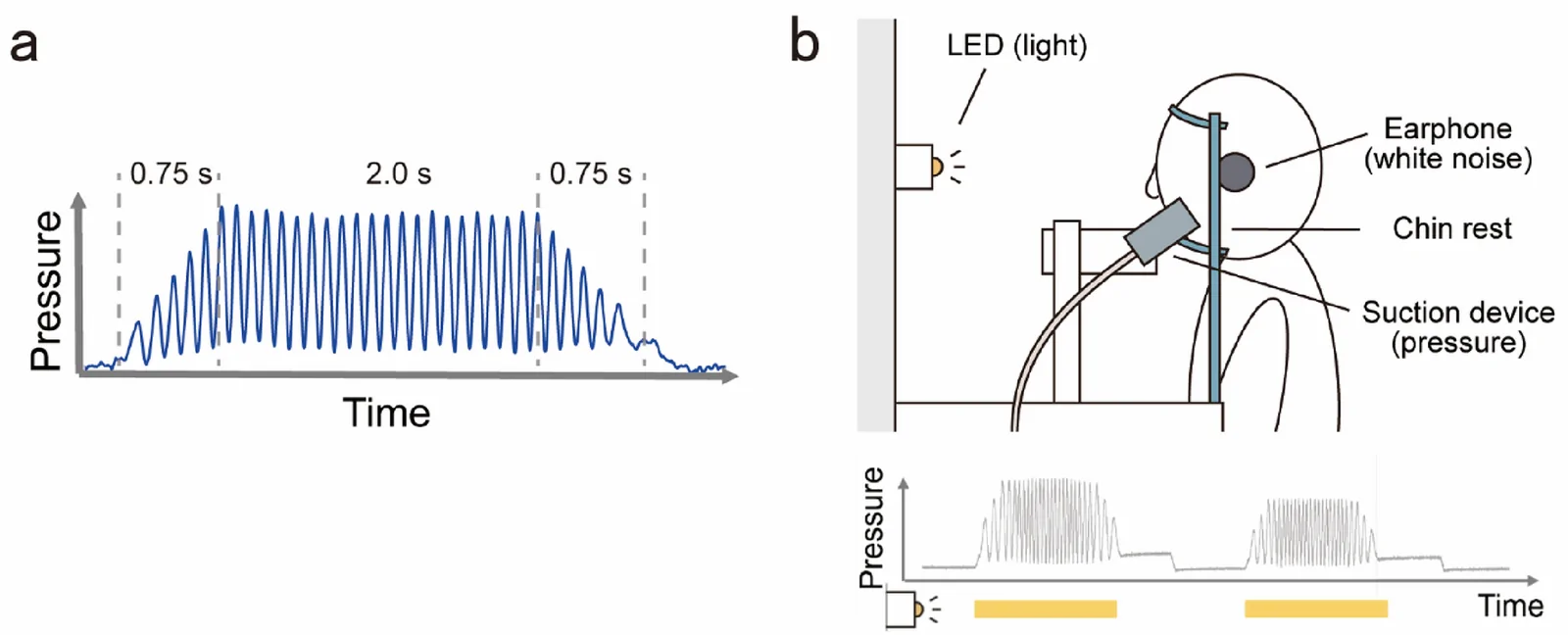
Tactile sensitivity was measured by oscillating suction stimuli on the cheek. (**a**) Example of a negative pressure stimulus during threshold measurement. Negative pressure intensity (kPa) versus time (s). The stimulus duration was 3.5 s, with 10 Hz stimulation for 2.0 s. (**b**) Experimental setup during threshold measurement. The participants positioned their faces on a chin rest to maintain consistent cheek placement. To minimize auditory impact on tactile perception, participants wore earphones that played white noise. An LED light indicated the presence of a stimulus.

Tactile sensitivity to negative pressure applied to the participants’ cheeks was quantitatively evaluated using psychophysical methods (Fig. 3). Each participant was asked to place her face on a chin rest to ensure contact between the contactor and the cheek. The participants were instructed to raise their hands upon sensing the stimulus.

We employed 10 Hz oscillation stimuli, which are more readily perceived by the skin than steady-state stimuli and correspond to a frequency at which sensory nerves terminating in Merkel cells and Meissner corpuscles exhibit high response sensitivity^59^. The use of dynamic vibrating stimuli, which are easier to perceive than static stimuli are, enabled us to evaluate a wide range of tactile sensory characteristics in participants (aged 26–61 years) via the same index without causing pain. The stimulus duration was 3.5 s, and within that time frame, 10 Hz stimulation was applied for 2.0 s (Fig. 3a). The range of the stimulus intensity was set from -0.05 to -12.3 kPa.

The tactile threshold was determined via the parameter estimation by sequential testing (PEST) method^60^, a psychophysical method that has been used to evaluate tactile thresholds. The PEST method increases the accuracy of the tactile threshold measurements while minimizing the number of measurements by adaptively changing the step of the presented stimulus on the basis of the participant’s responses. The method of determining the change in the stimulus was based on the four following rules. 1) If the direction of the stimulus step change was reversed, the step size was halved. 2) If the direction of the stimulus step remained constant, the step size was held constant. 3) If the direction of the stimulus step changed and remained constant for three consecutive steps, the step size was doubled. 4) If the stimulus step change remained constant for two consecutive steps, the step size change, whether maintained or doubled, depended on the previous reversal of the stimulus direction. Specifically, if the most recent reversal was due to doubling the step size, the current step size remained the same. Otherwise, the step size was doubled.

The measurement process started at the greatest stimulus intensity of -12.3 kPa. The stimulus intensity decreased if the participant perceived the stimulus and increased if the participant did not perceive the stimulus. The measurement ended when the step fluctuation was less than 0.1 kPa or when the upper or lower stimulus intensity limit was reached three consecutive times. If the step fluctuation range did not converge after a maximum of 30 trials, the final presented stimulus was used as the tactile threshold.

The measurements were performed three times for each participant. Considering the influence of skin fatigue due to repeated stimulation on the mechanical properties of the skin, three trials were conducted by shifting the stimulus presentation point by 5 mm on the cheek, and the interval between trials was at least 3 minutes. Practice trials were conducted before starting the test, and the measurements were acquired after the participants fully understood the stimulation procedure.

### Measurement of psychophysical characteristics

As part of the physiological measurements, height and weight were measured, and the body mass index (BMI) was calculated. Grip strength measurements were recorded for both hands.

Skin measurements included the assessment of the mechanical properties of the skin at the test site using a Cutometer^®^ (Courage & Khazaka Electronic GmbH) to evaluate skin rigidity and viscoelasticity.

Questionnaires were used to evaluate physical and mental states. The types of questionnaires used included the POMS2 (assesses mood states), the CMI (measures mental and physical health), the World Health Organization (WHO)-QOL26 (measures subjective well-being and QOL), the International Physical Activity Questionnaire (IPAQ) (a standardized questionnaire for evaluating physical activity levels), the Pittsburgh Sleep Quality Index (PSQI) (assesses the degree of sleep disturbances), and a questionnaire capturing ultraviolet exposure history.

### Analysis

The negative pressure data measured were processed via MATLAB software (MathWorks, R2023b). The relationship between negative pressure and tactile response was evaluated for each stimulus trial. For the tactile threshold values obtained, the median of three trials was used as the representative threshold for each participant. All the statistical analyses were performed in R (version 4.1.3) using the packages lme4, lmerTest, emmeans, and exactRankTests.

Associations between the continuous predictor threshold and each outcome variable were analyzed using linear mixed-effects models to account for the nonindependence of observations within twin pairs. The threshold was included as a fixed-effect predictor, and twin pair was included as a random intercept. For each outcome variable, the regression coefficient for the threshold (β), standard error, degrees of freedom, t value, and p value were extracted from the fitted model. Ninety-five percent confidence intervals for regression coefficients were estimated using Wald intervals derived from the fitted models. Because multiple outcome variables were examined, p values were adjusted using false discovery rate correction on the basis of the Benjamini–Hochberg procedure. Standardized regression coefficients were calculated by scaling β using the standard deviations of the predictor and outcome variables and were used as measures of effect size. Effect sizes were interpreted using conventional benchmarks for standardized effects (0.1 small, 0.3 medium, 0.5 large)^61^. Associations were considered relevant when the FDR-adjusted p value was < 0.05 and the standardized regression coefficient indicated a large effect size (|β_std| ≥ 0.50) (Fig. 1**; **Supplementary Table S1).

Paired comparisons between twins with lower and higher thresholds within each pair were analyzed using the Wilcoxon signed-rank test. Data are presented as the median (IQR) for each condition, and the median paired difference (high − low) was calculated. Effect sizes were expressed as matched-pairs rank-biserial correlations calculated from the signed ranks of the nonzero paired differences. Two-sided exact p values were calculated for the paired comparisons. For exploratory purposes, associations were considered relevant if *p* < 0.10 and *r* > 0.5 (Fig. 2**; **Table 2**; **Supplementary Table S2).

Group differences between twins from pairs in which both twins had low thresholds and pairs in which both twins had high thresholds were analyzed using linear mixed-effects models with twin pair included as a random intercept to account for within-pair correlation. Group (low vs. high) was included as a fixed effect. Estimated marginal means were calculated, and contrasts between groups (high − low) were obtained. The results are reported as the mean ± SD together with model-based estimated marginal means. The group difference (β), 95% confidence interval, and p value were calculated for each outcome variable. Statistical significance was defined as *p* < 0.05 (Fig. 2**; **Supplementary Table S3).

## Additional Information

The authors declare that they have no conflicts of interest.

## Supplementary Information

Supplementary Information.

## Data Availability

The small number of twin participants, coupled with the unique identifying nature of the twin status, facilitates easier identification of individuals. Therefore, owing to the Center for Twin Research’s policies to protect individual’s privacy, the data have not been made publicly accessible. However, upon reasonable request, data may be obtained with the permission of the Center for Twin Research, the University of Osaka Graduate School of Medicine (research@twin.med.osaka-u.ac.jp).
