# Supplementary material for: Reduced sensitivity to tactile stimuli associated with physical and mental disorders: A monozygotic twin study
Source: Sci Rep. 2026 Jun 10;16:26398. doi: 10.1038/s41598-026-56098-0 (PMC13500838; doi:10.1038/s41598-026-56098-0)
Supplement: Supplementary file 1 [file 41598_2026_56098_MOESM1_ESM.docx]

Supplementary Figure S1. UV exposure history

UV exposure history was assessed using a self-reported score indicating the average time spent in sunlight on a typical summer holiday (0: almost none, 1: ~1 h, 2: ~2 h, …, 6: ≥6 h). For each pair, the difference in the UV exposure score was calculated by subtracting the score of the lower-threshold twin from that of the higher-threshold twin. Positive values indicate greater UV exposure in the higher-threshold twin, whereas negative values indicate greater UV exposure in the lower-threshold twin; for example, “+4” indicates that the higher-threshold twin spent approximately 4 hours longer per day in sunlight.

Supplementary Table S1. Associations between the tactile threshold and outcome variables estimated by linear mixed-effects models

Regression coefficients (β), standardized effect sizes (β_std), standard errors (SE), degrees of freedom (df), t statistic (t), 95% confidence intervals (CI_low, CI_high), p values (p), and FDR-adjusted p values (p_fdr) for associations between the tactile threshold and each outcome variable, estimated using linear mixed-effects models with twin pair as a random intercept.

| Variable | β | β_std | SE | df | t | CI_low | CI_high | p | p_fdr |
| --- | --- | --- | --- | --- | --- | --- | --- | --- | --- |
| CMI_Heart | 0.915 | 0.394 | 0.308 | 48 | 2.97 | 0.311 | 1.519 | 0.005 | 0.292 |
| CMI_Physical | 0.417 | 0.362 | 0.164 | 47.72 | 2.548 | 0.096 | 0.738 | 0.014 | 0.444 |
| POMS2_FI | 0.267 | 0.32 | 0.113 | 47.738 | 2.376 | 0.047 | 0.488 | 0.022 | 0.444 |
| POMS2_F | 0.306 | 0.303 | 0.14 | 39.573 | 2.18 | 0.031 | 0.58 | 0.035 | 0.444 |
| CMI_Digestive | 0.47 | 0.288 | 0.229 | 46.65 | 2.051 | 0.021 | 0.919 | 0.046 | 0.451 |
| POMS2_CB | 0.195 | 0.278 | 0.099 | 41.218 | 1.966 | 0.001 | 0.389 | 0.056 | 0.451 |
| POMS2_TMD | 0.952 | 0.275 | 0.5 | 45.443 | 1.902 | -0.029 | 1.932 | 0.064 | 0.451 |
| CMI_Psychological | 0.676 | 0.261 | 0.373 | 47.571 | 1.814 | -0.055 | 1.408 | 0.076 | 0.451 |
| POMS2_DD | 0.159 | 0.256 | 0.087 | 40.039 | 1.817 | -0.012 | 0.33 | 0.077 | 0.451 |
| PSQIG | 0.152 | 0.255 | 0.085 | 47.529 | 1.788 | -0.015 | 0.318 | 0.08 | 0.451 |
| POMS2_AH | 0.2 | 0.238 | 0.121 | 47.742 | 1.656 | -0.037 | 0.436 | 0.104 | 0.469 |
| CMI_Anxiety | 0.831 | 0.233 | 0.498 | 47.933 | 1.668 | -0.146 | 1.808 | 0.102 | 0.469 |
| CMI_Sensitivity | 0.866 | 0.215 | 0.587 | 46.621 | 1.475 | -0.284 | 2.015 | 0.147 | 0.495 |
| CMI_Anger | 0.011 | 0.212 | 0.008 | 44.21 | 1.453 | -0.004 | 0.026 | 0.153 | 0.495 |
| R1 | 0.001 | 0.206 | 0.001 | 47.989 | 1.453 | 0 | 0.002 | 0.153 | 0.495 |
| CMI_Respiratory | 0.301 | 0.2 | 0.221 | 46.011 | 1.362 | -0.132 | 0.735 | 0.18 | 0.495 |
| CMI_Depression | 0.476 | 0.2 | 0.337 | 48 | 1.413 | -0.184 | 1.136 | 0.164 | 0.495 |
| CMI_Habit | 0.877 | 0.198 | 0.627 | 48 | 1.398 | -0.352 | 2.107 | 0.168 | 0.495 |
| POMS2_TA | 0.143 | 0.19 | 0.11 | 46.289 | 1.294 | -0.073 | 0.359 | 0.202 | 0.495 |
| CMI_Nervousness | 0.465 | 0.19 | 0.363 | 45.68 | 1.283 | -0.246 | 1.177 | 0.206 | 0.495 |
| R4 | 0.001 | 0.189 | 0.001 | 47.969 | 1.336 | -0.001 | 0.003 | 0.188 | 0.495 |
| CMI_Urogenital | 0.535 | 0.179 | 0.442 | 47.414 | 1.21 | -0.331 | 1.401 | 0.232 | 0.504 |
| grip_left | 0.135 | 0.158 | 0.11 | 45.071 | 1.23 | -0.08 | 0.35 | 0.225 | 0.504 |
| CMI_DiseaseFrequency | 0.225 | 0.118 | 0.271 | 47.955 | 0.83 | -0.306 | 0.757 | 0.41 | 0.808 |
| CMI_Fatigue | 0.352 | 0.084 | 0.613 | 40.784 | 0.575 | -0.849 | 1.554 | 0.568 | 0.895 |
| IPAQ_Strong | 0.572 | 0.074 | 1.111 | 48 | 0.515 | -1.606 | 2.751 | 0.609 | 0.936 |
| CMI_Musculoskeletal | 0.161 | 0.066 | 0.364 | 45.946 | 0.441 | -0.553 | 0.875 | 0.661 | 0.946 |
| IPAQ_Middle | 0.267 | 0.059 | 0.654 | 48 | 0.408 | -1.016 | 1.55 | 0.685 | 0.946 |
| CMI_EyeEar | 0.149 | 0.058 | 0.379 | 46.417 | 0.393 | -0.594 | 0.892 | 0.696 | 0.946 |
| CMI_Maladjustment | 0.203 | 0.057 | 0.512 | 39.404 | 0.396 | -0.802 | 1.207 | 0.694 | 0.946 |
| IPAQ_ModerateIntensity | 2.112 | 0.051 | 6.06 | 41.585 | 0.348 | -9.765 | 13.988 | 0.729 | 0.946 |
| IPAQ_VigorousIntensity | 6.677 | 0.048 | 20.901 | 45.321 | 0.319 | -34.288 | 47.642 | 0.751 | 0.946 |
| R6 | 0.001 | 0.042 | 0.003 | 44.584 | 0.321 | -0.005 | 0.007 | 0.75 | 0.946 |
| WQOL_3Social | 0.006 | 0.041 | 0.02 | 47.689 | 0.295 | -0.034 | 0.046 | 0.769 | 0.95 |
| CMI_Skin | 0.099 | 0.03 | 0.489 | 47.332 | 0.203 | -0.858 | 1.057 | 0.84 | 0.962 |
| R0 | 0 | 0.029 | 0.002 | 43.921 | 0.225 | -0.003 | 0.003 | 0.823 | 0.962 |
| grip_right | 0.026 | 0.027 | 0.122 | 43.008 | 0.215 | -0.213 | 0.265 | 0.831 | 0.962 |
| CMI_MedicalHistory | 0.021 | 0.016 | 0.186 | 48 | 0.113 | -0.344 | 0.386 | 0.911 | 0.993 |
| R3 | 0 | 0.014 | 0.002 | 44.601 | 0.108 | -0.003 | 0.003 | 0.914 | 0.993 |
| R9 | 0 | 0.009 | 0 | 43.269 | 0.059 | 0 | 0 | 0.953 | 1 |
| POMS2_VA | 0.01 | 0.009 | 0.178 | 42.767 | 0.057 | -0.339 | 0.36 | 0.955 | 1 |
| IPAQ_Categories | 0 | -0.002 | 0.023 | 43.053 | -0.013 | -0.045 | 0.044 | 0.989 | 1 |
| CMI_Nervous | -0.036 | -0.024 | 0.207 | 47.473 | -0.172 | -0.441 | 0.37 | 0.864 | 0.972 |
| R8 | 0 | -0.037 | 0.001 | 38.125 | -0.335 | -0.003 | 0.002 | 0.74 | 0.946 |
| IPAQ_Total | -12.123 | -0.048 | 37.944 | 43.88 | -0.32 | -86.492 | 62.245 | 0.751 | 0.946 |
| WQOL_2Psycho | -0.01 | -0.091 | 0.016 | 40.987 | -0.622 | -0.041 | 0.021 | 0.537 | 0.89 |
| IPAQ_Walking | -20.949 | -0.096 | 32.31 | 44.551 | -0.648 | -84.277 | 42.378 | 0.52 | 0.886 |
| WQOL_4Enviromental | -0.013 | -0.097 | 0.019 | 45.493 | -0.653 | -0.051 | 0.025 | 0.517 | 0.886 |
| WQOL_Ave | -0.011 | -0.114 | 0.014 | 43.101 | -0.767 | -0.039 | 0.017 | 0.447 | 0.829 |
| WQOL_1Physical | -0.016 | -0.136 | 0.016 | 48 | -0.95 | -0.048 | 0.017 | 0.347 | 0.705 |
| R7 | -0.002 | -0.14 | 0.002 | 36.858 | -1.338 | -0.006 | 0.001 | 0.189 | 0.495 |
| R2 | -0.003 | -0.15 | 0.002 | 39.699 | -1.321 | -0.007 | 0.001 | 0.194 | 0.495 |
| R5 | -0.003 | -0.158 | 0.002 | 41.201 | -1.341 | -0.008 | 0.001 | 0.187 | 0.495 |
| DEQS_Score | -0.412 | -0.169 | 0.355 | 41.978 | -1.16 | -1.108 | 0.284 | 0.252 | 0.53 |
| WQOL_5All | -0.025 | -0.187 | 0.019 | 45.424 | -1.266 | -0.063 | 0.014 | 0.212 | 0.495 |
| IPAQ_work | -2.897 | -0.253 | 1.648 | 43.254 | -1.758 | -6.126 | 0.333 | 0.086 | 0.451 |

Supplementary Table S2. Within-pair comparisons between twins with lower and higher tactile thresholds

Results of paired comparisons within twin pairs using the Wilcoxon signed-rank test, including median (IQR), median paired differences (high − low), rank-biserial correlation effect sizes (r), and exact p values (p).

|  | Lower threshold |  | Higher threshold |  |  |  |  |
| --- | --- | --- | --- | --- | --- | --- | --- |
| Variable | Median | IQR | Median | IQR | Median difference | p | Effect size (r) |
| CMI_Physical | 5.6 | 2.8 | 14.2 | 5.2 | 6.2 | 0.016 | 1 |
| CMI_Digestive | 3.6 | 1.8 | 10.7 | 7.1 | 7.1 | 0.062 | 0.905 |
| POMS2_TMD | 10 | 6 | 22 | 20.5 | 13 | 0.047 | 0.893 |
| POMS2_FI | 2 | 2.5 | 5 | 3.5 | 3 | 0.094 | 0.857 |
| POMS2_AH | 3 | 1 | 5 | 6 | 2 | 0.078 | 0.786 |
| CMI_Psychological | 2 | 11.8 | 19.6 | 9.8 | 15.7 | 0.078 | 0.786 |
| CMI_Urogenital | 15.4 | 15.4 | 23.1 | 7.7 | 15.4 | 0.094 | 0.75 |
| R2 | 0.7 | 0.1 | 0.6 | 0.1 | 0 | 0.094 | -0.714 |
| CMI_Musculoskeletal | 10 | 15 | 20 | 10 | 10 | 0.125 | 1 |
| R7 | 0.3 | 0.1 | 0.3 | 0.1 | 0 | 0.125 | -0.867 |
| CMI_Heart | 7.1 | 14.3 | 21.4 | 17.9 | 7.1 | 0.125 | 0.762 |
| CMI_Habit | 14.3 | 21.4 | 42.9 | 28.6 | 28.6 | 0.141 | 0.643 |
| POMS2_CB | 3 | 1.5 | 4 | 3 | 1 | 0.156 | 0.714 |
| POMS2_TA | 4 | 1 | 5 | 6 | 3 | 0.172 | 0.643 |
| CMI_Fatigue | 0 | 14.3 | 28.6 | 21.4 | 14.3 | 0.219 | 0.619 |
| R1 | 0.1 | 0 | 0.1 | 0 | 0 | 0.219 | 0.571 |
| R4 | 0.1 | 0.1 | 0.1 | 0 | 0 | 0.219 | 0.571 |
| CMI_Anxiety | 0 | 5.6 | 11.1 | 22.2 | 0 | 0.25 | 1 |
| CMI_Skin | 11.1 | 16.7 | 11.1 | 38.9 | 0 | 0.25 | 0.8 |
| CMI_Sensitivity | 0 | 0 | 16.7 | 33.3 | 0 | 0.25 | 0.8 |
| WQOL_5All | 3.5 | 0.8 | 3 | 0.5 | -0.5 | 0.25 | -0.733 |
| POMS2_DD | 1 | 1.5 | 2 | 3 | 0 | 0.25 | 0.6 |
| CMI_Anger | 0 | 0.1 | 0.1 | 0.4 | 0.1 | 0.25 | 0.571 |
| CMI_Nervousness | 0 | 0 | 11.1 | 5.6 | 11.1 | 0.281 | 0.524 |
| WQOL_1Physical | 2.7 | 0.3 | 2.4 | 0.4 | -0.3 | 0.312 | -0.524 |
| CMI_Maladjustment | 0 | 12.5 | 16.7 | 20.8 | 16.7 | 0.312 | 0.5 |
| grip_left | 23.5 | 2.5 | 25 | 1.8 | 0.5 | 0.344 | 0.524 |
| CMI_EyeEar | 10 | 5 | 20 | 10 | 10 | 0.375 | 0.6 |
| R5 | 0.4 | 0 | 0.4 | 0.1 | 0 | 0.438 | -0.429 |
| R0 | 0.3 | 0.1 | 0.3 | 0 | 0 | 0.469 | 0.357 |
| R3 | 0.3 | 0.1 | 0.4 | 0 | 0 | 0.469 | 0.357 |
| DEQS_Score | 10 | 12.5 | 6.7 | 10.8 | -3.3 | 0.469 | -0.357 |
| POMS2_F | 7 | 2 | 9 | 3 | 1 | 0.469 | 0.321 |
| POMS2_VA | 5 | 3.5 | 3 | 3.5 | 0 | 0.5 | -0.4 |
| CMI_MedicalHistory | 0 | 3.3 | 6.7 | 3.3 | 6.7 | 0.562 | 0.4 |
| WQOL_Ave | 3.2 | 0.4 | 2.8 | 0.5 | 0 | 0.578 | -0.286 |
| CMI_Respiratory | 0 | 11.9 | 4.8 | 16.7 | 0 | 0.625 | 0.4 |
| WQOL_4Enviromental | 3.6 | 0.6 | 3.6 | 0.9 | 0 | 0.688 | -0.238 |
| CMI_Nervous | 5.3 | 7.9 | 5.3 | 5.3 | 5.3 | 0.688 | 0.238 |
| IPAQ_Total | 693 | 1317.8 | 577.5 | 559.5 | 165 | 0.688 | -0.214 |
| IPAQ_work | 30 | 57.5 | 20 | 12.5 | 0 | 0.812 | -0.2 |
| R6 | 0.4 | 0.1 | 0.4 | 0.1 | 0 | 0.812 | 0.143 |
| PSQIG | 6 | 3 | 6 | 2.5 | 1 | 0.812 | 0.143 |
| WQOL_2Psycho | 2.8 | 0.3 | 3 | 0.3 | -0.2 | 0.812 | -0.143 |
| grip_right | 27.5 | 3.3 | 26.5 | 3.3 | -1.5 | 0.922 | -0.071 |
| IPAQ_Categories | 0 | 1 | 0 | 1 | 0 | 1 | -0.333 |
| IPAQ_VigorousIntensity | 0 | 200 | 0 | 120 | 0 | 1 | -0.1 |
| IPAQ_Walking | 198 | 1064.3 | 396 | 387.8 | 132 | 1 | -0.048 |
| R8 | 0.2 | 0 | 0.2 | 0 | 0 | 1 | 0 |
| R9 | 0 | 0 | 0 | 0 | 0 | 1 | 0 |
| IPAQ_ModerateIntensity | 0 | 40 | 0 | 60 | 0 | 1 | 0 |
| IPAQ_Strong | 0 | 25 | 0 | 15 | 0 | 1 | 0 |
| IPAQ_Middle | 0 | 10 | 0 | 15 | 0 | 1 | 0 |
| WQOL_3Social | 3.3 | 0.3 | 3.3 | 0.5 | 0 | 1 | 0 |
| CMI_DiseaseFrequency | 0 | 0 | 0 | 5.6 | 0 | 1 | 0 |
| CMI_Depression | 0 | 8.3 | 0 | 16.7 | 0 | 1 | 0 |

Supplementary Table S3. Between-pair comparisons of outcome variables in the low- and high-threshold twin groups

Group differences between twin pairs in which both twins had low thresholds and those in which both had high thresholds, estimated using linear mixed-effects models with twin pair as a random intercept. The number of observations in each group (n_Low, n_High), the group means (mean_Low, mean_High), the standard deviations (sd_Low, sd_High), estimated marginal means (emmean_Low, emmean_High), group differences (β), 95% confidence intervals (CI_low, CI_high), and p values (p) are reported.

| Variable | n_Low | n_High | mean_Low | sd_Low | mean_High | sd_High | emmean_Low | emmean_High | β | CI_low | CI_high | p |
| --- | --- | --- | --- | --- | --- | --- | --- | --- | --- | --- | --- | --- |
| CMI_Physical | 16 | 20 | 10.455 | 5.167 | 9.537 | 5.159 | 10.455 | 9.537 | -0.918 | -5.486 | 3.65 | 0.676 |
| CMI_Digestive | 16 | 20 | 9.598 | 9.287 | 8.571 | 6.596 | 9.598 | 8.571 | -1.027 | -8.505 | 6.451 | 0.775 |
| CMI_Psychological | 16 | 20 | 10.417 | 10.685 | 13.039 | 13.854 | 10.417 | 13.039 | 2.623 | -8.601 | 13.847 | 0.627 |
| CMI_Urogenital | 16 | 20 | 16.827 | 13.535 | 13.846 | 14.891 | 16.827 | 13.846 | -2.981 | -15.068 | 9.106 | 0.608 |
| POMS2_TMD | 16 | 20 | 8 | 11.243 | 11.95 | 20.179 | 8 | 11.95 | 3.95 | -9.953 | 17.853 | 0.555 |
| POMS2_AH | 16 | 20 | 3.625 | 3.442 | 4.45 | 4.685 | 3.625 | 4.45 | 0.825 | -2.866 | 4.516 | 0.642 |
| POMS2_FI | 16 | 20 | 4.25 | 2.62 | 5.85 | 4.859 | 4.25 | 5.85 | 1.6 | -2.133 | 5.333 | 0.377 |
| R2 | 16 | 20 | 0.649 | 0.08 | 0.625 | 0.081 | 0.649 | 0.625 | -0.024 | -0.102 | 0.053 | 0.517 |
